# Ethnic differences in smoking intensity and COPD risk: an observational study in primary care

**DOI:** 10.1038/s41533-017-0052-8

**Published:** 2017-09-04

**Authors:** Alexander Gilkes, Sally Hull, Stevo Durbaba, Peter Schofield, Mark Ashworth, Rohini Mathur, Patrick White

**Affiliations:** 1 Department of Primary Care and Public Health Sciences, King’s College London, London, UK; 2 Queen Mary, Centre for Primary Care and Public Health, University of London, London, UK

## Abstract

Chronic obstructive pulmonary disease risk is lower in black and south Asian people than white people, when adjusting for age, sex, deprivation and smoking status. The role of smoking intensity was assessed for its contribution to ethnic differences in chronic obstructive pulmonary disease risk, a relationship not previously investigated. This cross-sectional study included routinely collected primary care data from four multi-ethnic London boroughs. Smoking intensity (estimated by cigarettes per day) was compared between ethnic groups. Chronic obstructive pulmonary disease risk was compared between ethnic groups using multiple logistic regression, controlling for age, sex, deprivation, asthma and both smoking status and smoking intensity, examined independently. In all, 1,000,388 adults were included. Smoking prevalence and intensity were significantly higher in the white British/Irish groups than other ethnic groups. Higher smoking intensity was associated with higher chronic obstructive pulmonary disease risk. Chronic obstructive pulmonary disease risk was significantly lower in all ethnic groups compared with white British/Irish after adjustment for either smoking status or smoking intensity, with lowest risk in black Africans (odds ratio 0.33; confidence interval 0.28–0.38). Ethnic differences in chronic obstructive pulmonary disease risk were not explained in this study by ethnic differences in smoking prevalence or smoking intensity. Other causes of ethnic differences in chronic obstructive pulmonary disease risk should be sought, including ethnic differences in smoking behaviour, environmental factors, repeated respiratory infections, immigrant status, metabolism and addictiveness of nicotine and differential susceptibility to the noxious effects of cigarette smoke.

## COPD: Smoking intensity not behind ethnic differences in disease risk

Lower smoking intensity among blacks and south Asians does not explain their lower risk for chronic obstructive pulmonary disease (COPD). A UK team led by Alexander Gilkes from Kings College London analysed primary care data from more than a million people living in four multi-ethnic boroughs of the British capital. The researchers found that smoking status and intensity (as measured by number of cigarettes smoked per day) were both significantly higher in white British or Irish groups than in other ethnic populations. Even after statistically adjusting for smoking status or smoking intensity, however, the researchers couldn’t account for the fact that people of south Asian or African descent had much lower prevalence rates of COPD, a lung disease linked to smoking. The findings suggest that other explanations of ethnic differences are still needed.

## Introduction

Chronic obstructive pulmonary disease (COPD) is an important healthcare burden in the UK in terms of deaths, healthcare costs and hospital admissions.^1, 2^ The major risk factor for COPD in the UK is smoking. Previous studies in London have identified that both black (adjusted odds ratio [OR] 0.44; 95% confidence interval [CI] 0.39 to 0.51) and south Asian (OR 0.82; CI 0.68 to 0.98) populations had a lower risk of COPD compared to white populations when adjusting for age, sex, deprivation and smoking status.^3, 4^ Whether severity of COPD varies by ethnicity is unclear. It appears to depend partly on whether adjustment is made for ethnicity in predicted values for forced expiratory volume in 1 second (FEV_1_).^3^ In both of the above studies lower rates of smoking in black and south Asian groups were accounted for in COPD risk analyses.

US surveys have suggested that black people are more likely to be smokers, but smoke fewer cigarettes per day (CPD), based on number of packs per day.^5, 6^ The Health Survey for England 2004 assessed smoking status and intensity by ethnic group, with sampling designed to boost sample size in minority groups. Compared to 24% of the general male population, 21% of black African and 40% of Bangladeshi men reported smoking. Two percent of Bangladeshi women and 24% of black Caribbean women smoked compared to 23% of women overall. Thirty-one percent of male smokers smoked 20 or more CPD, ranging from 5% in black African men to 33% in white Irish men. Twenty-seven percent of female smokers smoked 20 or more per day.^7^ Overall smoking prevalence in England has dropped steadily from 26% in 2003^8^ to 16.9% in 2015.^9^ CPD measures one dimension of smoking intensity or tobacco exposure. It has the advantage of being recorded routinely in UK general practice, but it does not account for cumulative exposure, depth of inhalation or puffs per cigarette.^10, 11^

The proportion of UK hospital admissions attributable to smoking is decreasing, although the absolute number among over 35s is increasing. The number of UK deaths estimated to be caused by smoking remains large at 78,200 (17%) in 2013.^8^

This study was set in the London boroughs of Hackney, Lambeth, Newham and Tower Hamlets, which include some of the most multi-ethnic and socially deprived areas in England.^12^ Using routinely-collected clinical data from primary care we aimed to determine if differences in smoking prevalence and intensity as measured by CPD were associated with gender and ethnicity, and to determine whether ethnic differences in smoking intensity explained ethnic differences in COPD risk. This relationship has not previously been examined in a population study.

## Results

### Participants

In all, 1,000,388 GP-registered adults were included. Summary characteristics of the population are shown in Table 1 and of the 13,703 people with a COPD diagnosis in Table 2. Ethnicity was recorded for 89% of the population and 96% of COPD patients. Crude prevalence of GP-recorded COPD diagnosis was 1.4% and prevalence of spirometry-confirmed COPD diagnosis was 0.7%.

**Table 1:** Smoking history and COPD diagnosis in ethnic groups in London (*n* = 1,000,388)

Ethnic group	Number	Mean age years	Male %	Mean IMD score	Asthma diagnosis %	Never smoker %^a^	Current smoker %^a^	Ex-smoker %^a^	Ever-smokers with CPD recorded %	Recorded COPD diagnosis %	Spirometry-confirmed COPD %
White British	234,642	44	49	36	15	45	26	27	57	3.2	1.8
White Irish	13,429	45	51	36	11	41	28	28	54	4.2	2.2
Other white	179,899	37	46	37	6	47	29	20	59	0.8	0.3
Black Caribbean	45,355	49	43	39	14	53	24	21	61	1.5	0.7
Black African	75,058	41	47	41	6	78	9	11	52	0.3	0.1
Other Black	34,260	40	46	42	12	62	21	14	68	0.6	0.2
Indian	58,082	39	56	38	8	78	11	8	67	0.8	0.3
Pakistani	34,415	37	61	39	9	74	15	8	73	0.8	0.3
Bangladeshi	95,356	37	55	44	9	65	21	11	79	0.9	0.4
Other Asian	27,795	39	51	36	9	71	14	12	60	0.6	0.2
Mixed/Chinese/other	79,234	39	45	38	8	62	19	15	59	0.7	0.3
Unknown/not stated	113,863	38	58	36	7	50	21	14	54	0.5	0.2
All	1,000,388	40	50	38	10	56 (562,640)	22 (219,934)	18 (177,885)	60	1.4	0.7

^a^ Smoking statuses do not add up to 100% because smoking status was unknown in a small proportion of patients

**Table 2:** Smoking history and spirometry-confirmed diagnosis among people with COPD in ethnic groups in London (*n* = 13,703)

Ethnic group	Number	Mean age years	Male %	Mean IMD score	Asthma diagnosis %	Never smoker %^a^	Current smoker %^a^	Ex-smoker %^a^	Ever-smokers with CPD recorded %	Spirometry-confirmed COPD %
White British	7804	68	49	41	30	8	43	49	72	55
White Irish	568	68	54	38	30	7	43	49	68	51
Other white	1355	64	57	40	29	13	42	45	69	43
Black Caribbean	692	71	63	39	39	20	32	48	61	46
Black African	241	59	60	41	37	49	16	33	58	29
Other Black	207	58	57	43	41	37	32	29	76	32
Indian	437	67	68	38	44	46	24	30	69	33
Pakistani	257	63	74	40	44	37	23	38	70	36
Bangladeshi	894	64	77	45	32	24	31	45	71	40
Other Asian	174	62	70	36	43	33	26	40	65	39
Mixed/Chinese/other	515	64	61	40	38	28	32	39	69	40
Unknown/not stated	559	66	60	37	27	17	36	44	60	35
All	13,703	67	55	40	32	14 (1877)	39 (5157)	46 (6070)	70	49

^a^ Smoking statuses do not add up to 100% because smoking status was unknown in a small proportion of patients

### Smoking history

Overall, smoking status was recorded for 96% of the population, and CPD was recorded for 82% of current smokers and 33% of ex-smokers. Among COPD patients, smoking status was recorded for 99.4% and CPD was recorded for 95% of current smokers and 49% of ex-smokers.

Recording of smoker status was between 96 and 99% in all ethnic groups in London, except in those in whom ethnicity was unknown (Table 3). Over half of white British and white Irish people had ever smoked. A fifth of black African and Indian people had ever smoked. People in all ethnic groups other than white Irish were significantly less likely to have ever smoked than white British, ranging from OR 0.21 (CI 0.21 to 0.22) for the Indian group, to OR 0.90 (CI 0.89 to 0.91) for the other white group. Rates of ever smoking were significantly higher in men than women in all ethnic groups, this gender difference being particularly pronounced in the south Asian ethnic groups.

**Table 3:** Smoking prevalence and intensity (cigarettes per day, CPD) by ethnic group and sex (*n* = 1,000,388)

Ethnicity (*n*)	Smoker status recorded %	Male ever smokers				Female ever smokers			
		Ever smokers (% of males)	% Ever smokers with CPD	Mean CPD	% Heavy smokers (20 + CPD)	Ever smokers (% of females)	% Ever smokers with CPD	Mean CPD	% Heavy smokers (20 + CPD)
White British (234,642)	98	66,931 (56)	55	11.4	24	61,538 (50)	60	9.6	17
White Irish (13,249)	97	4128 (61)	53	12.0	28	3400 (51)	54	9.2	16
White (179,899)	97	45,135 (55)	58	11.3	23	43,702 (45)	60	8.0	10
Black Caribbean (45,355)	99	11,478 (58)	60	7.5	9	9129 (36)	62	6.8	6
Black African (75,058)	98	10,684 (30)	52	7.7	9	4189 (11)	50	6.4	5
Other black (34,260)	97	6926 (44)	68	7.8	9	5122 (28)	69	6.8	6
Indian (58,082)	97	9394 (29)	68	6.8	6	1842 (7)	63	5.7	4
Pakistani (34,415)	97	7172 (34)	74	7.9	10	952 (7)	69	6.6	6
Bangladeshi (95,356)	97	27,445 (52)	79	8.0	8	3843 (9)	74	5.5	3
Other Asian (27,795)	98	5318 (37)	62	7.7	9	2044 (15)	56	6.3	6
Mixed/Chin/other (79,234)	96	15,667 (44)	60	9.2	15	11,529 (27)	59	7.2	8
Unknown (113,863)	85	25,681 (38)	55	9.9	17	14,570 (31)	53	8.1	11
All (1,000388)	96	235,959 (47)	61	9.7	17	161,860 (33)	60	8.3	12

Crude mean CPD was higher in the white British (10.5 CPD), white Irish (10.7 CPD) and other white (9.6 CPD) ethnic groups than all other ethnic groups (range 6.6 to 8.4). Women smoked fewer CPD than men in all ethnic groups (see Table 3).

### COPD risk

COPD risk was significantly higher for the white Irish group (OR 1.19; CI 1.05 to 1.34) and significantly lower for all other ethnic groups compared to the white British group (Table 4). Lowest COPD risk was among black Africans (OR 0.33; CI 0.28 to 0.38). Smoking status and smoking intensity had almost identical influences on the COPD risk of individual ethnic groups (Table 4).

**Table 4:** Comparison of COPD risk for different ethnic groups adjusting for (i) smoking status or (ii) smoking intensity

COPD risk	(i) Adjusting for smoking status (*n* = 856,090)^a^		(ii) Adjusting for smoking intensity (*n* = 717,253)^a^	
	OR	95% CI	OR	95% CI
White British	1		1	
White Irish	1.19	1.05 to 1.34	1.15	1.02 to 1.31
Other white	0.59	0.53 to 0.66	0.58	0.52 to 0.64
Black Caribbean	0.39	0.35 to 0.43	0.38	0.33 to 0.42
Black African	0.33	0.28 to 0.38	0.38	0.32 to 0.44
Other Black	0.40	0.35 to 0.47	0.44	0.37 to 0.53
Indian	0.71	0. 62 to 0.82	0.71	0. 61 to 0.83
Pakistani	0.73	0.63 to 0.84	0.71	0.61 to 0.83
Bangladeshi	0.64	0.58 to 0.72	0.60	0.53 to 0.68
Other Asian	0.56	0.47 to 0.67	0.57	0.47 to 0.70
Chinese/mixed/other	0.50	0.44 to 0.55	0.52	0.46 to 0.58

^a^ Multiple logistic regression adjusting for age, sex, deprivation, asthma, practice clustering and either (i) smoking status (never, current, ex-smoker) or (ii) smoking intensity (never, light, moderate, heavy smoker)

Smoking intensity was associated with progressively higher COPD risk than never smoking, for light smokers (OR 9.9; CI 9.0 to 10.9), moderate smokers (OR 12.1; CI 11.0 to 13.3) and heavy smokers (OR 16.4; CI 14.6 to 18.5) when adjusting for age, gender, socio-economic status, asthma and ethnicity.

### COPD risk among never smokers

The rates of never having smoked among COPD patients were highest among black African/Caribbean and south Asian groups (Table 2). Black African COPD patients were over 17 times more likely (OR 17.6; CI 13.1 to 23.6) to have never smoked than white British COPD patients. Results were similar among spirometry-confirmed COPD patients (see Supplementary Table 1 for full results).

To follow up the striking ethnic differences in never smoker rates among COPD patients, we performed a further analysis of COPD risk on the whole population stratified by ever/never smoker status. Among never smokers, COPD risk was lowest in black Caribbeans (OR 0.60; CI 0.48 to 0.75) and black Africans (OR 0.63; CI 0.50 to 0.79) and highest in white Irish (OR 1.41; CI 1.03 to 1.34), compared to the white British group. COPD risk among never smokers in all other ethnic groups was not significantly different to white British. Among ever smokers, COPD risk across ethnic groups was similar to the main analysis in Table 4, with the exception of black Africans, in whom COPD risk was even lower (OR 0.22; CI 0.18 to 0.26) (see Supplementary Table 2 for full results).

### Sensitivity analyses for diagnostic accuracy and missing smoking intensity

#### Accurate diagnosis

The outcome of assessment of risk of spirometry-confirmed COPD (rather than simply labelled COPD) did not significantly differ from the results of the main logistic regression analysis in Table 4, except for the white Irish group. In this sub-analysis, the risk of spirometry-confirmed COPD in the white Irish group was no different to the white British group. All other ethnic groups still had significantly lower risk than white British. This was true for both smoking status and smoking intensity analyses (see Supplementary Table 3 for full results).

#### Missing smoking intensity data

The outcome of assessment of COPD risk when replacing missing smoking intensity data with either never smoker, light smoker, moderate smoker, or heavy smoker in turn was not significantly different from the main logistic regression analysis in Table 4. COPD risk for people in all minority ethnic groups remained lower than white British, except for the white Irish group, which still had higher risk (see Supplementary Table 4 for full results).

## Discussion

### Main findings

People from ethnic minority groups in London were less likely to smoke and were lighter smokers than the majority white British and the white Irish groups. COPD risk based on GP recording was significantly lower in all minority ethnic groups compared to the white British and white Irish groups. Adjusting for smoking intensity (as measured by numbers of CPD) rather than smoking status did not significantly affect the relationship between ethnicity and COPD risk. Therefore, ethnic differences in COPD prevalence were not explained by differences in smoking intensity. Sensitivity analyses using spirometry-confirmed COPD and imputing values for missing smoking intensity data gave very similar results. This suggests that misdiagnosis and missing smoking intensity data did not influence this risk estimate.

Our data suggest that white British and white Irish people are more susceptible to COPD than other ethnic groups. Other causes of ethnic differences in risk of COPD should be sought. These might include biological differences in the metabolism and addictive potential of nicotine, ethnic differences in smoking behaviour (e.g., number of inhalations per cigarette, depth of inhalation), biological differences in susceptibility to the noxious effects of tobacco smoke and different exposure or susceptibility to other environmental risk factors.

We observed markedly different rates of never having smoked between COPD patients of different ethnic groups. This suggests that causes other than direct smoking are important in COPD pathogenesis.

### Interpretation of findings in relation to previously published work

The finding of significantly lower COPD risk among minority ethnic groups is consistent with our previous paper.^3^ The current study included a much larger population with a more varied ethnic makeup. This significantly added to the previous study by allowing more robust associations, particularly by ethnic subgroup rather than ethnic group. Numbers in each ethnic subgroup were sufficient to allow adjustment of analyses for smoking intensity.

Bruse et al.^13^ previously examined the effect of smoking intensity on the relationship between COPD risk and Hispanic or non-Hispanic white ethnicity among 1949 recruited smokers and ex-smokers. They also found that higher rates of smoking were associated with greater COPD risk but differences in smoking rates did not explain the ethnic differences in COPD risk. The current study is based on a representative population sample from four urban geographical areas. It includes a broad ethnic mix, and it has determined COPD status by the presence of a diagnosis of COPD rather than screening spirometry.

The prevalence of GP-diagnosed COPD of 1.4% in this study is significantly lower than estimates obtained in epidemiological studies that use spirometry screening to determine COPD diagnosis.^14^ It has been previously shown that only around half of COPD in England is diagnosed.^15^ However, most undiagnosed cases are likely to represent asymptomatic mild disease that does not require treatment,^16^ and Global Initiative for Chronic Obstructive Lung Disease (GOLD) does not advocate screening asymptomatic individuals.^17^

### Strengths and limitations of this study

This study examined a large data set of over one million GP registered adults in multi-ethnic areas of London. The large sample size allowed robust analysis and yielded estimates with narrow CIs. The sensitivity analyses suggested that misdiagnosis and missing smoking intensity data were unlikely to have affected the results. This was the first population study to assess the role of smoking intensity in the ethnic risk of COPD. Because data were routinely collected during clinical encounters, we could not independently verify the data quality, although implausible values were removed.

CPD measures only one dimension of smoking exposure and intensity. It cannot measure inhalation rate, depth of inhalation and total inhalations per cigarette. We used the most recently recorded value for CPD in this study. This was similar to the mean CPD for individuals, suggesting that CPD is a stable measure over time. CPD may be subject to “digit bias” with patients likely to report consumption in units of 10.^10^ This effect was apparent in the multi-modal distribution of the CPD variable, with peaks at 1, 5, 10, 15 and 20 CPD (data not shown in this paper).

A measure such as pack-years smoked could be used to assess the role of cumulative tobacco exposure in the risk of COPD, and may be more closely correlated with disease risk.^18^ Pack-years smoked were not recorded in this data set and could not be calculated from available variables.

Differences in the way cigarettes are smoked (inhalation volume, inhalations per cigarette, duration of inhalation), as well as brand and type of cigarette, can significantly affect the dose of nicotine and harmful substances per cigarette, further explaining the disease risk.^19^ In addition, there is some evidence that ethnic differences in the way cigarettes are smoked may make CPD a worse predictor of carcinogen exposure in US black smokers.^6^ This relationship may be different in the more ethnically diverse population of London. Subtle differences in inhalation patterns are difficult to accurately measure at the individual level, even in the laboratory setting. CPD is likely to be the best measure of smoking intensity currently routinely available in large numbers at population level.

There were no routinely recorded data available on cannabis or shisha smoking. These activities may be associated with lung disease including COPD,^20, 21^ and may be more prevalent in certain ethnic groups.^22^

The ethnic groupings used in the UK have limitations when it comes to extrapolating our findings to other societies.^23^ While our findings may not be directly translatable internationally where different ethnic categorisations are used, they do highlight the large ethnic differences in COPD risk. These differences should be investigated in other communities to better understand healthcare inequalities, the variable risks of smoking and the pathogenesis of COPD.

Country of birth and length of residency in the UK probably vary by ethnicity and it is likely that they were additional factors in the association between ethnicity and risk of COPD. We did not have data on either of these factors.

There is significant variation in normal spirometry between ethnic groups and the use of ethnic-specific normal spirometry values remains controversial. We addressed this in our previous paper.^3^ The use of ethnic-specific normal spirometry values mainly influences the assessment of disease severity rather than diagnosis and risk. COPD risk was the focus of this paper.

### Implications for future research, policy and practice

Further research is required into other underlying causes of the ethnic differences in COPD risk. This should include examining other aspects of smoking patterns and intensity by ethnic group in the UK, such as number of puffs per cigarette, nicotine absorption per cigarette and nicotine metabolism and addictiveness, as well as ethnic variation in healthcare use, healthcare-seeking behaviour, variation in disease detection and variation in susceptibility to the noxious effects of cigarette smoke. There may be genetic or epigenetic reasons for some of these ethnic differences, but it is important not to equate ethnicity with genetics.^23^

An unexpected finding was that never smokers made up a significantly higher proportion of COPD patients in ethnic minorities, especially black Africans, when compared to white British. Nonetheless, black Africans who had never smoked were still significantly less likely (OR 0.63; CI 0.50 to 0.79) to have COPD than white British people who had never smoked. It is possible that there could be differences between ethnic groups in exposure to COPD risk factors other than direct smoking, such as uncontrolled asthma, biomass smoke, occupational exposure, repeated respiratory tract infections and passive smoking. Some of these could be related to country of birth and length of residency in the UK, which is likely to vary between ethnic groups. More detailed evaluation of these factors should be undertaken in COPD patients of all ethnicities who have never smoked.

Smoking prevalence and intensity varied significantly between ethnic groups in London. These differences may be useful to inform public health smoking cessation strategies.

## Conclusions

White British and Irish people in London are more likely to smoke and smoke more CPD than people in other ethnic groups. Although smoking intensity is an important factor in COPD risk, it did not explain the significant ethnic differences in COPD risk. The underlying cause remains unclear.

## Methods

### Study design, setting and sample

This was a cross-sectional study using anonymised, routinely collected primary care data from Hackney, Lambeth, Newham and Tower Hamlets. Data were collected with the approval of the Information Governance Committees from each London borough Clinical Commissioning Group. The anonymised data were managed and stored in accordance with UK NHS information governance requirements. Ethical approval was not required for the use of these anonymised data. We reported this study according to the STROBE guidelines.^24^ All adults aged over 18 registered with participating practices were included. Data were extracted on 31 October 2013.

### Variables

Demographic data included age, sex and socioeconomic deprivation. Deprivation was measured at patient level by the Index of Multiple Deprivation (IMD-10) score, and mapped to lower super-output area using postcode. IMD score is based on national census and local authority data, and reflects deprivation specific to geographical areas.^12^

Ethnicity was self-reported and recorded by practices according to UK 2001 Census categories.^25^ We grouped together the small subgroups of Chinese, mixed, and other. We used 11 ethnic groupings as follows: white British; white Irish; other white; black Caribbean; black African; other black; Indian; Pakistani; Bangladeshi; other Asian; Chinese/mixed/other; unknown/not stated. Subgroups were reported in order to reveal significant differences within the same ethnic group if they were large enough to allow comparative analysis.

Clinical variables included diagnostic Read codes for asthma and for COPD. All patients with a GP-recorded diagnosis of COPD (including emphysema, chronic bronchitis and other ICD-ten diagnostic codes J41–J44.9 consistent with COPD) were included. This represented symptomatic disease and differs from true prevalence. A spirometry-confirmed COPD subgroup was identified. It comprised patients with a GP-recorded COPD diagnosis and confirmation of obstructive spirometry, that is, ratio of FEV_1_/FVC < 0.7, as defined by GOLD.^17^ Where necessary the FEV_1_/FVC ratio was calculated from the latest recorded FEV_1_ and FVC values.

Smoking status was assigned as never smoker, current smoker, ex-smoker or unknown. Where there were conflicting older smoking codes in the same patient, the most appropriate code was used. For example, most recent code “never smoked tobacco” or “current non-smoker” with any previous positive smoking history became “ex-smoker”. Any patients with a CPD value but no smoking status recorded were re-classified as “current smoker”. The most recent value for CPD was extracted, where recorded. We considered using maximum and mean patient values for CPD, but found no significant difference between them. Because the CPD variable had a multimodal distribution, patients were assigned a smoking intensity category to enable statistical analysis. Smoking intensity categories were: never smoker; light smoker (0–9 CPD); moderate smoker (10–19 CPD); heavy smoker (20 + CPD). Where there were multiple values for a variable, the most recent was used, unless specified otherwise.

### Bias

The data set included all adults registered with a general practitioner, which in England comprises more than 97% of the population.^26^ Therefore, the data set was likely to be representative of the population, with a low risk of bias. There was a risk of bias due to missing smoking intensity data. Therefore, a sensitivity analysis using imputed missing CPD values was performed, see below.

### Statistical methods

Baseline population characteristics and crude COPD and spirometry-confirmed COPD prevalence were calculated overall and for each ethnic group. For each ethnic group, raw mean CPD and adjusted mean CPD (adjusting for age, sex and deprivation using linear regression) were calculated. The main analysis compared COPD risk between the ethnic groups using multiple logistic regression, controlling for age, sex, deprivation, asthma and either smoking status (never, current, ex-smoker) or smoking intensity (never, light, moderate, heavy smoker). A supplementary analysis repeated the main analysis, stratified by smoking status. Among COPD patients, probability of currently smoking and of being a never smoker were compared between ethnic groups. All logistic regression analyses were adjusted for clustering at practice level, using robust standard errors. Patients with unknown or not stated ethnicity were excluded from analyses. Stata software version 14 (StataCorp LP, College Station, TX, USA) was used for all statistical analysis.

### Sensitivity analyses

#### Accurate diagnosis

To assess whether associations were influenced when only spirometry-confirmed COPD diagnoses were included, logistic regression analyses were performed with spirometry-confirmed COPD as the dependent variable.

#### Missing smoking intensity data

To assess whether ever-smokers with and without CPD values were fundamentally different due to different recording of CPD between ethnic groups, the smoking intensity logistic regression analyses were repeated with imputed values for missing smoking intensity data. All missing smoking intensity values were replaced with either (i) never smoker, (ii) light smoker, (iii) moderate smoker or (iv) heavy smoker. These included the most extreme possible scenarios for the missing data.

### Ethical approval

Ethical approval was not required as patient-level data were anonymised and aggregated patient data were reported in this study. All GPs in the participating east and south London practices consented to the use of anonymised patient data for research and development for patient benefit.

### Data availability

Data used for this study can be made available on written request, subject to information governance requirements being met.

## Electronic supplementary material

Supplementary Tables 1 to 4
