# Supplementary material for: Ethnic differences in smoking intensity and COPD risk: an observational study in primary care
Source: NPJ Prim Care Respir Med. 2017 Sep 4;27:50. doi: 10.1038/s41533-017-0052-8 (PMC5583254; doi:10.1038/s41533-017-0052-8)
Supplement: Supplementary file 1 — Supplementary Tables 1 to 4 [file 41533_2017_52_MOESM1_ESM.docx]

**Supplementary Table 1: Comparison of probability of never smoking among (i) all COPD patients and (ii) spirometry-confirmed COPD patients.**

| Probability of never smoking | (i) All COPD patients  (n = 12,999)* | | (ii) Spirometry-confirmed COPD patients (n = 6,416)* | |
| --- | --- | --- | --- | --- |
|  | OR | 95% CI | OR | 95% CI |
| White British | 1 |  | 1 |  |
| White Irish | 1.0 | 0.7 to 1.4 | 1.3 | 0.7 to 2.2 |
| Other white | 2.0 | 1.6 to 2.5 | 2.2 | 1.4 to 3.4 |
| Black Caribbean | 3.6 | 2.8 to 4.7 | 3.2 | 2.2 to 4.9 |
| Black African | 17.6 | 13.1 to 23.6 | 19.5 | 11.3 to 33.5 |
| Other black | 9.0 | 6.3 to 12.7 | 8.9 | 5.1 to 15.6 |
| Indian | 14.9 | 11.3 to 19.5 | 16.6 | 10.3 to 26.5 |
| Pakistani | 11.5 | 8.7 to 15.2 | 13.3 | 7.5 to 23.6 |
| Bangladeshi | 6.2 | 4.9 to 7.9 | 6.2 | 4.3 to 8.9 |
| Other Asian | 8.9 | 5.4 to 14.7 | 14.5 | 7.1 to 29.6 |
| Chinese/mixed/other | 6.1 | 4.7 to 8.0 | 5.8 | 3.7 to 9.2 |
| * Multiple logistic regression adjusting for age, sex, deprivation, asthma and practice clustering. | | | | |

**Supplementary Table 2: Comparison of COPD risk for different ethnic groups stratified by smoker status.**

| COPD risk | (i) Never smokers  (n = 501,571)* | | (ii) Ever smokers  (n = 354,519)* | |
| --- | --- | --- | --- | --- |
|  | OR | 95% CI | OR | 95% CI |
| White British | 1 |  | 1 |  |
| White Irish | 1.41 | 1.02 to 1.94 | 1.17 | 1.03 to 1.34 |
| Other white | 0.83 | 0.67 to 1.04 | 0.57 | 0.51 to 0.63 |
| Black Caribbean | 0.60 | 0.48 to 0.75 | 0.36 | 0.33 to 0.40 |
| Black African | 0.63 | 0.50 to 0.79 | 0.22 | 0.18 to 0.26 |
| Other black | 0.95 | 0.73 to 1.22 | 0.31 | 0.26 to 0.37 |
| Indian | 1.07 | 0.87 to 1.32 | 0.60 | 0.51 to 0.71 |
| Pakistani | 1.06 | 0.84 to 1.34 | 0.63 | 0.53 to 0.76 |
| Bangladeshi | 1.13 | 0.92 to 1.40 | 0.55 | 0.49 to 0.63 |
| Other Asian | 0.84 | 0.62 to 1.16 | 0.48 | 0.40 to 0.59 |
| Chinese/mixed/other | 0.90 | 0.75 to 1.08 | 0.43 | 0.37 to 0.49 |
| * Multiple logistic regression adjusting for age, sex, deprivation, asthma and practice clustering, stratified by smoker status. | | | | |

**Supplementary Table 3: Sensitivity Analysis 1: accounting for accurate diagnosis**

**Comparison of ethnic risk of (i) diagnosed COPD or (ii) spirometry-confirmed COPD, adjusting for smoking status (n = 856,090).**

| COPD risk | (i) COPD* | | (ii) spirometry-confirmed COPD* | |
| --- | --- | --- | --- | --- |
|  | OR | 95% CI | OR | 95% CI |
| White British | 1 |  | 1 |  |
| White Irish | 1.19 | 1.05 to 1.34 | 1.06 | 0.90 to 1.26 |
| Other white | 0.59 | 0.53 to 0.66 | 0.50 | 0.42 to 0.58 |
| Black Caribbean | 0.39 | 0.35 to 0.43 | 0.35 | 0.31 to 0.40 |
| Black African | 0.33 | 0.28 to 0.38 | 0.21 | 0.16 to 0.27 |
| Other black | 0.40 | 0.35 to 0.47 | 0.28 | 0.21 to 0.36 |
| Indian | 0.71 | 0. 62 to 0.82 | 0.47 | 0. 38 to 0.59 |
| Pakistani | 0.73 | 0.63 to 0.84 | 0.54 | 0.43 to 0.67 |
| Bangladeshi | 0.64 | 0.58 to 0.72 | 0.47 | 0.39 to 0.58 |
| Other Asian | 0.56 | 0.47 to 0.67 | 0.46 | 0.34 to 0.62 |
| Chinese/mixed/other | 0.50 | 0.44 to 0.55 | 0.40 | 0.35 to 0.46 |
| * Multiple logistic regression adjusting for age, sex, deprivation, smoking status, asthma, practice clustering. | | | | |

**Comparison of ethnic risk of (i) diagnosed COPD or (ii) spirometry-confirmed COPD, adjusting for smoking intensity (n = 717,253).**

| COPD risk | (i) COPD* | | (ii) spirometry-confirmed COPD* | |
| --- | --- | --- | --- | --- |
|  | OR | 95% CI | OR | 95% CI |
| White British | 1 |  | 1 |  |
| White Irish | 1.15 | 1.02 to 1.31 | 1.10 | 0.92 to 1.31 |
| Other white | 0.58 | 0.52 to 0.64 | 0.49 | 0.41 to 0.58 |
| Black Caribbean | 0.38 | 0.33 to 0.42 | 0.33 | 0.28 to 0.39 |
| Black African | 0.38 | 0.32 to 0.44 | 0.22 | 0.17 to 0.30 |
| Other black | 0.44 | 0.37 to 0.53 | 0.31 | 0.22 to 0.42 |
| Indian | 0.71 | 0. 61 to 0.83 | 0.44 | 0.34 to 0.56 |
| Pakistani | 0.71 | 0.61 to 0.83 | 0.48 | 0.38 to 0.62 |
| Bangladeshi | 0.60 | 0.53 to 0.68 | 0.38 | 0.30 to 0.47 |
| Other Asian | 0.57 | 0.47 to 0.70 | 0.45 | 0.32 to 0.65 |
| Chinese/mixed/other | 0.52 | 0.46 to 0.58 | 0.40 | 0.34 to 0.46 |
| * Multiple logistic regression adjusting for age, sex, deprivation, smoking intensity, asthma, practice clustering. | | | | |

**Supplementary Table 4: Sensitivity analysis 2: accounting for missing smoking intensity data.**

**Comparison of ethnic risk of COPD when replacing missing smoking intensity data with (i) never smoker; (ii) light smoker; (iii) moderate smoker; (iv) heavy smoker.**

| COPD risk | Raw smoking intensity data  (n = 717,253) | | (i) Missing becomes never smoker  (n = 856,090) | | (ii) Missing becomes light smoker  (n = 856,090) | | (iii) Missing becomes moderate smoker  (n = 856,090) | | (iv) Missing becomes heavy smoker  (n = 856,090) | |
| --- | --- | --- | --- | --- | --- | --- | --- | --- | --- | --- |
|  | OR | 95% CI | OR | 95% CI | OR | 95% CI | OR | 95% CI | OR | 95% CI |
| White British | 1 |  | 1 |  | 1 |  | 1 |  | 1 |  |
| White Irish | 1.15 | 1.02 to 1.31 | 1.23 | 1.09 to 1.39 | 1.20 | 1.07 to 1.36 | 1.19 | 1.06 to 1.35 | 1.20 | 1.06 to 1.36 |
| Other white | 0.58 | 0.52 to 0.64 | 0.59 | 0.54 to 0.65 | 0.61 | 0.55 to 0.67 | 0.60 | 0.54 to 0.66 | 0.59 | 0.53 to 0.65 |
| Black Caribbean | 0.38 | 0.33 to 0.42 | 0.37 | 0.33 to 0.41 | 0.44 | 0.40 to 0.48 | 0.40 | 0.36 to 0.44 | 0.38 | 0.34 to 0.42 |
| Black African | 0.38 | 0.32 to 0.44 | 0.28 | 0.25 to 0.33 | 0.35 | 0.30 to 0.41 | 0.33 | 0.29 to 0.39 | 0.31 | 0.27 to 0.36 |
| Other black | 0.44 | 0.37 to 0.53 | 0.37 | 0.32 to 0.43 | 0.45 | 0.39 to 0.53 | 0.42 | 0.36 to 0.49 | 0.39 | 0.33 to 0.45 |
| Indian | 0.71 | 0. 61 to 0.83 | 0.52 | 0.45 to 0.59 | 0.76 | 0.67 to 0.87 | 0.70 | 0.61 to 0.81 | 0.66 | 0.58 to 0.76 |
| Pakistani | 0.71 | 0.61 to 0.83 | 0.56 | 0.49 to 0.65 | 0.79 | 0.68 to 0.91 | 0.73 | 0.63 to 0.84 | 0.68 | 0.59 to 0.78 |
| Bangladeshi | 0.60 | 0.53 to 0.68 | 0.56 | 0.50 to 0.62 | 0.73 | 0.66 to 0.82 | 0.64 | 0.57 to 0.71 | 0.58 | 0.52 to 0.65 |
| Other Asian | 0.57 | 0.47 to 0.70 | 0.48 | 0.40 to 0.57 | 0.61 | 0.52 to 0.72 | 0.58 | 0.49 to 0.68 | 0.54 | 0.45 to 0.64 |
| Chinese/mixed/other | 0.52 | 0.46 to 0.58 | 0.45 | 0.40 to 0.51 | 0.53 | 0.47 to 0.59 | 0.50 | 0.45 to 0.56 | 0.48 | 0.43 to 0.54 |
| * Multiple logistic regression adjusting for age, sex, deprivation, asthma, practice clustering and smoking status. Missing smoking status data replaced with either (i) never smoker; (ii) light smoker; (iii) moderate smoker; (iv) heavy smoker. | | | | | | | | | | |
